# OCTA Multilayer and Multisector Peripapillary Microvascular Modeling for Diagnosing and Staging of Glaucoma

**DOI:** 10.1167/tvst.9.2.58

**Published:** 2020-11-05

**Authors:** Danilo Andrade De Jesus, Luisa Sánchez Brea, João Barbosa Breda, Ella Fokkinga, Vera Ederveen, Noor Borren, Amerens Bekkers, Michael Pircher, Ingeborg Stalmans, Stefan Klein, Theo van Walsum

**Affiliations:** 1 Biomedical Imaging Group Rotterdam, Department of Radiology and Nuclear Medicine, Erasmus MC, Rotterdam, The Netherlands; 2 Research Group of Ophthalmology, Department of Neurosciences, KU Leuven, Leuven, Belgium; 3 Cardiovascular R&D Center, Faculty of Medicine of the University of Porto, Porto, Portugal; 4 Ophthalmology Department, Centro Hospitalar Sao João, Porto, Portugal; 5 Delft University of Technology, Delft, The Netherlands; 6 Center for Medical Physics and Biomedical Engineering, Medical University of Vienna, Vienna, Austria; 7 Ophthalmology Department, University Hospitals Leuven, Leuven, Belgium

**Keywords:** OCT angiography, microvascular density, glaucoma, machine learning

## Abstract

**Purpose:**

To develop and assess an automatic procedure for classifying and staging glaucomatous vascular damage based on optical coherence tomography angiography (OCTA) imaging.

**Methods:**

OCTA scans (Zeiss Cirrus 5000 HD-OCT) from a random eye of 39 healthy subjects and 82 glaucoma patients were used to develop a new classification algorithm based on multilayer and multisector information. The averaged circumpapillary retinal nerve fiber layer (RNFL) thickness was also collected. Three models, support vector machine (SVM), random forest (RF), and gradient boosting (xGB), were developed and optimized for classifying between healthy and glaucoma patients, primary open-angle glaucoma (POAG) and normal-tension glaucoma (NTG), and glaucoma severity groups.

**Results:**

All the models, the SVM (area under the receiver operating characteristic [AUROC] 0.89 ± 0.06), the RF (AUROC 0.86 ± 0.06), and the xGB (AUROC 0.85 ± 0.07), with 26, 22, and 29 vascular features obtained after feature selection, respectively, presented a similar performance to the RNFL thickness (AUROC 0.85± 0.06) in classifying healthy and glaucoma patients. The superficial vascular plexus was the most informative layer with the infero temporal sector as the most discriminative region of interest. No significant differentiation was obtained in discriminating the POAG from the NTG group. The xGB model, after feature selection, presented the best performance in classifying the severity groups (AUROC 0.76± 0.06), outperforming the RNFL (AUROC 0.67± 0.06).

**Conclusions:**

OCTA multilayer and multisector information has similar performance to RNFL for glaucoma diagnosis, but it has an added value for glaucoma severity classification, showing promising results for staging glaucoma progression.

**Translational Relevance:**

OCTA, in its current stage, has the potential to be used in clinical practice as a complementary imaging technique in glaucoma management.

## Introduction

Glaucoma is the leading cause of irreversible blindness, affecting over 80 million people worldwide.^1^ It is a chronic progressive optic neuropathy characterized by the thinning of the peripapillary retinal nerve fiber layer (RNFL) and optic disc cupping as a result of axonal and retinal ganglion cell loss.^2^^,^^3^ Glaucoma is an irreversible but preventable disease, which requires a management strategy involving risk stratification. Risk assessment is meant to properly allocate intensive treatment and monitoring to those who are more vulnerable while avoiding overburdening both patient and health care system in cases when the disease is less likely to cause loss of visual function. However, the limited understanding of the disease's pathophysiology hampers effective risk stratification. Glaucoma assessment is currently based on a set of risk factors, previously identified in epidemiologic studies (e.g., RNFL thickness and intraocular pressure [IOP]), but with limited predictability for the individual patient.^4^^-^^6^ Therefore, there is a need to identify additional risk factors/features that may contribute to improve this clinical decision process.

Differences in vascular parameters have been reported between glaucoma patients and healthy individuals, at ocular and systemic levels.^7^^,^^8^ A number of techniques, such as fluorescein angiography,^9^ laser speckle flowgraphy,^10^ laser Doppler flowmetry,^11^ and color Doppler imaging,^12^^,^^13^ have been used for the evaluation of ocular and retinal blood perfusion. With the recent introduction of optical coherence tomography angiography (OCTA), standard OCT imaging devices are now capable of analyzing retinal vascular flow and to link it to a number of ocular diseases.^10^^,^^14^ The application of OCTA to glaucoma has contributed to a more comprehensive assessment of the vascular supply role in the disease modulation. Significantly lower vessel density and blood flow index in the peripapillary area,^15^^-^^19^ optic disc,^15^^-^^24^ and macular area,^15^^,^^17^^,^^18^^,^^23^^-^^25^ have been observed in glaucoma eyes in comparison with normal eyes. For all these areas, the diagnostic abilities of the imaged features increased with the glaucoma severity.^23^^-^^26^ Moreover, it has been previously reported,^27^ that vascular parameters increase the ability to discriminate between types of glaucoma (primary open-angle glaucoma [POAG] and normal-tension glaucoma [NTG]), as parameters linked to vascular dysfunction are more prominent in NTG patients.

Although three-dimensional OCTA information is generated from OCT imaging, most studies have only investigated the vascular density in the superficial layers (above the inner plexiform layer). Only a few studies considered the choroidal layers, including the choriocapillaris. Kiyota et al.,^28^ found significantly lower OCTA parameters in the superficial choroid (0–70 µm below Bruch's membrane, including the choriocapillaris) of glaucoma eyes in comparison to healthy controls but not in the deep choroid (70–140 µm below Bruch's membrane). Two other studies reported no significant differences in choroidal results.^29^^,^^30^ In addition to the analysis of all information available in a given layer, studies have been conducted discriminating different sectors. Andrade De Jesus et al.,^19^ showed that the inferior and superior sectors of the peripapillary superficial layer present the most severe vascular damage in glaucoma individuals compared to healthy controls. A similar study was performed by Lommatzsch et al.,^31^ who observed that macular vessel density in both superficial and deep retinal vascular plexus in glaucomatous eyes was significantly lower than in healthy eyes, with the largest reduction found in the inferior macular sector. Rao et al.,^23^ showed that the area under the receiver operating characteristic (AUROC) of the averaged peripapillary vessel density was significantly larger than the AUROC of the average inside the optic disc or at the macula, with the inferotemporal sector as its best discriminator.

Despite the increasing number of studies on OCTA features for glaucoma diagnosis, the data are frequently conflicting and/or arising from small-scale studies. Therefore, the current results have not allowed researchers to reach conclusions on the added value of a vascular analysis in clinical practice. The vascular glaucomatous damage in the retinal layers, choriocapillaris, and choroid, and the advantage of the vascular parameters compared to structural parameters such as the RNFL thickness or the intraocular pressure, need additional research to determine their contribution to the risk assessment and staging of glaucoma disease. Hence, the aim of this study is to further contribute to the understanding of the vascular role of glaucoma in the retinal layers, choriocapillaris, and choroid, looking at the information available at each layer and also at specific sectors within a layer. To that end, this article describes the design and optimization of classification models based on different layers and sectors. The models are compared with the aim to infer whether the information from multiple regions of interest (ROIs) has an added value for both the diagnostic accuracy and the discrimination between types of glaucoma (NTG and POAG). In summary, this article was designed to further contribute to the understanding of the following points:

• How good is a model based on multilayer and multisector (MLS) OCTA data at classifying between glaucoma patients and healthy subjects?
• Is there an added value of using OCTA imaging features in comparison to RNFL thickness?
• Can OCTA imaging features be used to discriminate between POAG and NTG?
• Can OCTA imaging be used to discriminate between different glaucoma severity levels?
• Which OCTA imaging features have the highest discriminant power in diagnosing and staging glaucoma disease?

## Methods and Materials

**Figure 1.**
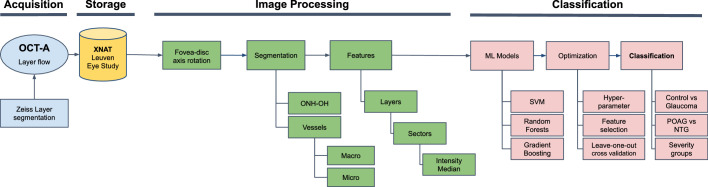
Study pipeline structured into four parts (*blue*: acquisition; *yellow*: storage; *green*: image processing; *red*: classification). ONH-OH, optic nerve head optically hollow area; ML, machine learning; SVM, support vector machine; POAG, primary open-angle glaucoma; NTG, normal-tension glaucoma.

The study pipeline was structured into four parts (acquisition, storage, image processing, and classification) as shown in Figure 1. In the first part, OCTA data were acquired by glaucoma specialists (J.B.B. and I.S.). Next, the data were exported to the Extensible Neuroimaging Archive Toolkit (XNAT) platform. Then, the imaged data were processed for masking out the spurious information and defining the different ROIs, in order to compute the respective microvascular density features. Lastly, two machine learning classification models were trained with the features obtained in the previous step. Feature selection was performed in order to gain insight into which features are the most important for risk stratification and disease staging.

### Image Acquisition

**Figure 2.**
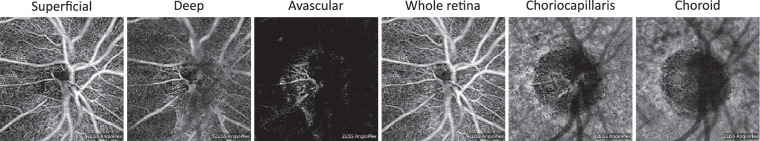
Optic disc centered peripapillary OCTA coronal projections of a healthy individual. From *left* to *right*: superficial, deep, avascular, whole retina, choriocapillaris, and choroid layers.

An OCTA data set selected from the Leuven Eye Study cohort,^12^ was used in this study. Thirty-nine healthy subjects (aged 63 ± 13 years) and 82 glaucoma patients (aged 69 ± 10 years with an average visual field mean deviation [VF MD] of –8.1 ± 6.7 dB) were included, as shown in Table 1. The OCTA data in the Leuven Eye Study cohort consist of, for each subject, a 3×3-mm optic disc centered angiography scan acquired (via undilated pupil) using the Cirrus 5000 HD OCT (Carl Zeiss, Dublin, CA, USA; 10.0 software version). In this cohort, healthy individuals were recruited from those accompanying glaucoma patients. The exclusion criteria included blood relatives, those with a family history of glaucoma, rim notching or thinning, optic disc structural changes such as asymmetrical cup/disc ratio, disc hemorrhage, or an IOP above 21 mm Hg. Patients with glaucoma were defined as having characteristic optic disc damage and visual field loss as defined elsewhere.^32^ Glaucoma patients were excluded if they had a history of ocular trauma or any other eye disease (including high ametropias, defined as hyperopia higher than 4 diopters and myopia higher than 6 diopters). Additionally, patients with diabetes mellitus were excluded, since it is a known confounder in vascular-related research. Patients with open-angle glaucoma were stratified based on their maximal recorded untreated IOP as having POAG (> 21 mm Hg) or NTG if equal or below that threshold. The eye with greater glaucomatous damage was chosen to be included in the study whenever both eyes were considered eligible. Six layers (superficial and deep vascular plexus, avascular, whole retina, choriocapillaris, and choroid) segmented by the manufacturer's software were exported (see Fig. 2). Although the avascular layer should not contain flow information, the glaucoma progression or the performance of the device's layer segmentation software may lead to microvasculature imaged in this layer, as seen in Figure 2. Therefore, the avascular layer was also included in order to maximize the information retrieved from the OCTA scan. Images with signal strength index below the suggested inclusion value provided by the manufacturer, 6 out of 10,^33^ were excluded from the study. Images with severe movement artifacts or visible floaters were also excluded. In addition to the angiography scans, the averaged circumpapillary RNFL thicknesses were exported from the device. The glaucoma group was further divided by severity. Patients with a VF MD higher than –6 dB were considered mild (37 subjects), between –6 and –12 dB were considered moderate (26 subjects), and those with a VF MD worse than –12 dB were considered severe (19 subjects). Visual fields were obtained using the Humphrey Field Analyzer (HFA) (Zeiss, Oberkochen, Germany) or the Octopus (Interzeag, Schlieren, Switzerland) perimeters on the same day as the OCT and OCTA examinations. The visual field programs were the 24-2 SITA standard program (HFA) or the G1 dynamic strategy (Octopus). The VF MD was extracted through a software that can extract data from both devices (Peridata; PeriData Software GmbH, Germany, version 3.5.4). Unreliable VFs (false positive >20%, false negative >40%, or fixation loss >30%) were excluded from analysis. These cutoffs are set by default by the manufacturer and widely used in previously published studies. The study adhered to the tenets of the Declaration of Helsinki, and it was approved by the Institutional Review Board of the University Hospitals Leuven. An informed consent was signed by all the participants prior to the study evaluation.

**Table 1.** Demographics and Characteristics of the Data Set for the Control and Glaucoma Groups, Type of Glaucoma, and Glaucoma Severity

				*Type of Glaucoma*			*Glaucoma Severity*			
	Control	Glaucoma	*p* Value	POAG	NTG	*p*-Value	Mild	Moderate	Severe	*p*-Value
Number of eyes	39	82		38	44		37	26	19	
Age (years)	62.2 ± 13.9	65.9 ± 8.9	0.10	64.4 ± 9.9	67.3 ± 7.8	0.20	66.3 ± 8.6	66.5 ± 7.7	64.6 ± 11.1	0.74
Gender (male/female)	24/15	41/41		18/20	23/21		13/24	17/9	11/8	
Eye (OD/OS)	20/19	47/35		17/21	30/14		19/18	16/10	12/7	
VF MD (dB)	0.38 ± 0.78	−7.8 ± 6.4	<0.001	−8.6 ± 7.4	−7.8 ± 6.4	0.49	−2.4 ± 2.4	−8.6 ± 1.7	−17.1 ± 4.1	<0.001
RNFL (µm)	88.9 ± 6.4	67.1 ± 12.2	<0.001	65.6 ± 12.2	68.4 ± 12.0	0.20	73.6 ± 11.8	63.3 ± 10.7	59.7 ± 7.2	<0.001
							73.6 ± 11.8	63.3 ± 10.7		0.002
							73.6 ± 11.8		59.7 ± 7.2	<0.001
								63.3 ± 10.7	59.7 ± 7.2	0.23
S-IT (au)	72.8 ± 17.2	33.1 ± 20.6	<0.001	33.5 ± 21.3	34.1 ± 19.7	0.8	47.1 ± 19.1	26.8 ± 16.1	17.5 ± 9.4	<0.001
							47.1 ± 19.1	26.8 ± 16.1		<0.001
							47.1 ± 19.1		17.5 ± 9.4	<0.001
								26.8 ± 16.1	17.5 ± 9.4	0.034

OD, right eye; OS, left eye; Age, VF MD, RNFL thickness, and S-IT are presented as mean ± standard deviation; S-IT, superficial inferotemporal microvascular density; au, arbitrary units.

### Storage and Database

All the acquisitions were exported via HyperText Transfer Protocol Secure (HTTPS) from a picture archiving and communication system (PACS) at University Hospitals Leuven to the Extensible Neuroimaging Archive Toolkit (XNAT,^34^) hosted within the Dutch national research infrastructure (Trait/Health-RI, https://trait.health-ri.nl/trait-tools/xnat). XNAT is an open-source platform for imaging-based research and clinical investigations, which manages access to different data sets compartmentalized into separate projects. All data were anonymized before being transferred to the XNAT.

### Image Processing

Python version 3.7, in combination with OpenCV,^35^ and Scikit-image,^36^ was used for the implementation of all the developed algorithms. The image processing was structured into three parts: fovea-disc axis correction, segmentation, and feature computation.

#### Fovea-Disc Axis Correction

**Figure 3.**
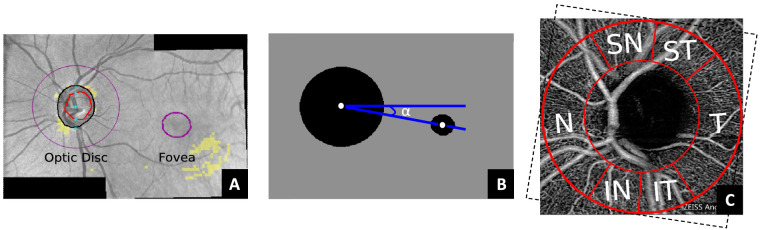
Example of a Panomap image used to estimate the fovea-disc axis angle (A). The binarization around the optic disc and the fovea, based on the predelineated areas by the device, is followed by the detection of the circles’ centroids and the estimation of the rotation angle (α) (B). The superimposed mask on the OCTA image at the optic disc (C) is rotated according to α. The Garway-Heath sectors nasal (N), inferonasal (IN), inferotemporal (IT), temporal (T), superotemporal (ST), and superonasal (SN) are delineated between the red lines.

First, the OCTA images were rotationally corrected to a common reference. This was applied to ensure that the same area was being compared during the sectorial analysis, as there are differences in the head position during OCTA acquisition. In this work, the Panomap images provided by the device (see Fig. 3A) were used to correct the angle of the fovea-disc axis to zero degrees. The Panomap provides colored circular delineations of the fovea and optic disc, which were used to estimate the angle between the centroids of both circles (Fig. 3B). The mask used to extract the ROIs in the OCTA image (explained in the following subsections) was rotated according to the estimated fovea-disc axis angle (α) and taking into account if the eye was a left (OS) or right (OD) eye (Fig. 3C).

#### Segmentation

**Figure 4.**
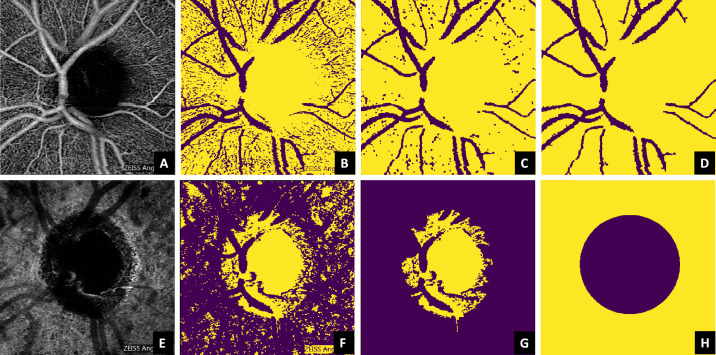
Segmentation of the microvasculature (*upper row*) and the optic nerve head optically hollow area (*lower row*) based on the superficial vascular plexus and the choroid, respectively. The steps from the original image until the respective mask are denoted from the *left* to the *right* and succinctly explained in Image Processing.

Two main structures of interest were segmented in order to be masked out during the feature computation: the macrovasculature and the optic nerve head optically hollow area (ONH-OH). The superficial layer (Fig. 4A) was chosen as an anchor reference for obtaining a macrovascular mask for all the layers, due its higher contrast in comparison to the other ones. For achieving an approximate estimate of the macrovasculature, a binary image (Fig. 4B) was obtained from the superficial OCTA layer, based on a threshold set by the upper 88th percentile of the image intensity histogram. The selection of the percentile was done empirically, and it may depend on the data set. The definition of a relative threshold (in terms of a percentile), rather than an absolute value, makes the method more robust to the variations of intensity expected among individuals. A morphologic opening followed by a closing was then applied to denoise the binary image (Fig. 4C). Next, all connected components with an area less than 250 pixels were removed, ensuring that the remaining area was macrovasculature/background (Fig. 4D).

**Figure 5.**
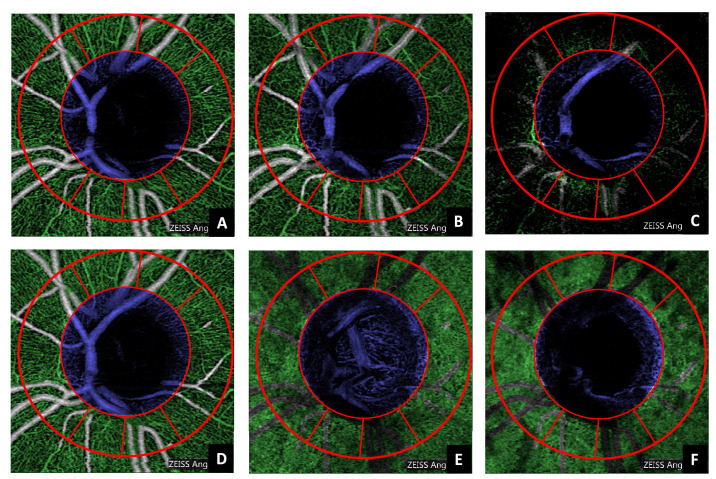
Automatic segmentation of the microvasculature (*green*) and the optic nerve head optically hollow area (*blue*) applied to all the layers included in this study (A, superficial vascular plexus; B, deep vascular plexus; C, avascular; D, whole retina; E, choriocapillaris; F, choroid). The *red lines* denote the Garway-Heath sectors already rotated according to the fovea-disc axis correction.

Regarding the ONH-OH, the choroid was used as anchor reference instead of the superficial layer. This is because, in some cases (Fig. 2), the center of the ONH-OH is partially covered by macrovasculature in the superficial layer, while the choroid offers a more robust alternative, as it provides a higher contrast between the ONH-OH and the vascularized areas (Fig. 4E). A binary image (Fig. 4F) was obtained from the choroidal OCTA image based on the lower 40th percentile of the equalized histogram. This percentile was empirically determined based on the data set characteristics. All connected components except the one with the largest area (estimated with Grana's algorithm for eight-way connectivity,^37^) were removed from the image (Fig. 4G). Its location and respective area were used to estimate the centroid and the radius of a circle, which was used as a mask for the optically hollow area (Fig. 4H). Even if the dimension of this region is anatomically dependent and may vary with the disease progression, the shape remains similar, allowing this generalization. Figure 5 illustrates the automatic segmentation applied to all the layers considered in this study.

#### Features

Once the vasculature and the optically hollow regions had been determined, the Garway-Heath map,^38^ was used to set the ROIs according to seven sectors: superotemporal (ST), superonasal (SN), nasal (N), inferonasal (IN), inferotemporal (IT), temporal (T), and the circumpapillary (CP) — all sectors included. A circular mask centered at the ONH-OH was created, ensuring that the dimension of the sectors was the largest possible, while keeping the radius constant for all of them. The last step of the image processing was to retrieve the microvascular density measurements (features) from the ROIs based on the microvascular intensity median (VIM) within each ROI. The median was chosen instead of the mean because it is more robust to outliers in the sample set, and it is more applicable to not normally distributed data. Hence, 42 features were obtained based on the information retrieved from six layers and seven sectors at the peripapillary region. For comparison purposes, the RNFL measure from the device was obtained for each patient. Additionally, the VIM measure for the IT sector of the superficial layer (S-IT) was used as a single-feature-based reference method, since it has been reported as the best glaucoma discriminator in the peripapillary region.^23^

### Classification

Python 3.7, in combination with Scikit-learn,^39^ and Numpy,^40^ was used for the classification step. Pandas,^41^ was used for data manipulation, and Matplotlib^42^ for obtaining the graphs and images for the results. The classification was organized into two parts: definition and training of the machine learning models, and statistical analysis and feature study, as described below.

#### Machine Learning Models

Three classification models were used for the experiments: a support vector machine (SVM),^43^ a random forest (RF),^44^ and a gradient boosting classifier (xGB).^45^ They were trained for three classification tasks: (1) differentiating glaucoma patients from controls, (2) distinguishing POAG and NTG types of glaucoma, and (3) identifying glaucoma severity levels (healthy, mild, moderate, and severe).

SVM was chosen because it typically provides robust classification performance even in case of small data sets and high-dimensional feature spaces. It is a supervised machine learning method that creates a hyperplane that separates the data points into two classes (negative and positive). The hyperplane is based on the maximal margin between classification points. The margin can be defined as the largest distance between nearest data points (the support vectors) and the hyperplane. Ideally, data points that are far apart are separable for classification, and that explains why the maximal margin is searched. The second model, RF, is a supervised machine learning model that is commonly used and relatively fast. It is an ensemble method that aggregates the individual predictions of decision trees into a combined prediction. RF creates several trees from subsets of the data, which are randomly selected. Each node in the tree represents a decision boundary that takes a certain amount of feature values into account. The features are also randomly divided as subsets for each tree. The obtained (random) trees are then merged to form the final class decision. The third model, xGB, is another supervised model that, like RF, consists of a set of decision trees. There are two main differences between these two models. First, RF builds each tree independently while gradient boosting uses the previous trees as a basis to build the subsequent ones. This additive model works in a forward stage wise manner, introducing a weak learner to improve the shortcomings of existing weak learners. Second, the RF combines results at the end of the process, while gradient boosting combines results along the way. For all models, one-versus-all strategy,^46^ was used for differentiating the glaucoma severity levels. One-versus-all involves training a single classifier per class, with the samples of that class as positive samples and all other samples as negatives.

**Figure 6.**
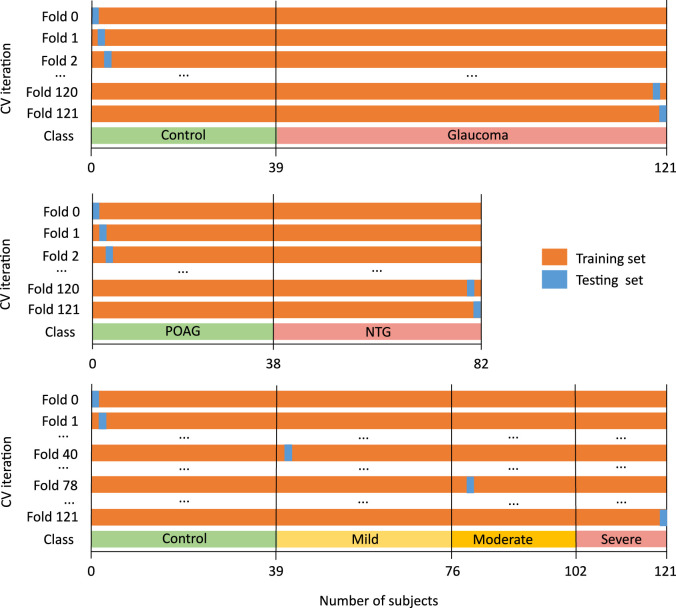
Leave-one-out cross-validation for differentiating glaucoma patients from controls (*first row*), distinguishing POAG and NTG types of glaucoma (*second row*), and identifying glaucoma severity levels (*third row*).

Leave-one-out cross-validation was used to split the data in 121 folds. This approach leaves one subject out of the training data; that is, if there are *n* data points in the original sample, then *n* – 1 samples are used to train the model and 1 point is used as the test set. Figure 6 summarizes the cross-validation scheme for each of the three classification tasks.

For each fold, hyperparameter optimization was applied on the training set. The hyperparameters of both classifiers were adjusted through grid search using stratified cross-validation,^47^ (5-folds) to prevent overfitting. Hyperparameters are parameters with specified values per classification model. They influence the accuracy, complexity, and computational time of the model. The following parameters were considered for the SVM: kernel type (linear or radial basis function); penalty parameter C of the error term $\in \{10^{-5},10^{-4},10^{-3},10^{-2},0.1,1,10,10^{2},10^{3}\}$; gamma coefficient for the radial basis function kernel $\in \{10^{-5},10^{-4},10^{-3},10^{-2},10^{-1},10^{0},10^{1},10^{2},10^{3}\}$. The grid search for the RF model included the following parameters: number of trees in the forest ∈{10,25,50}; function to measure the quality of a split (Gini index or entropy); whether bootstrap samples are used when building trees (yes, no); percentage (%) of the minimum number of samples required to be at a leaf node ∈{10,30,50}; percentage (%) of the minimum number of samples required to split ∈{10,30,50}; maximum depth of the tree ∈{5,10,15,unlimited}. The following parameters were included in the xGB grid search: minimum sum of instance weight (Hessian) needed in a child ∈{1,5,10}; step size shrinkage used in update to prevent overfitting (learning rate) ∈{0.01,0.1,0.25}; minimum loss reduction required to make a further partition on a leaf node of the tree ∈{0.1,1,3}; subsample ratio of the training instances ∈{0.6,0.8,1}; subsample ratio of columns when constructing each tree ∈{0.6,0.8,1}; percentage (%) of the minimum number of samples required to be at a leaf node ∈{10,30,50}; maximum depth of the tree ∈{5,10,15,unlimited}.

In order to study the contribution of different features to the model and to improve the diagnostic accuracy, feature selection and hyperparameter tuning were applied on the training set. Ideally, including more features in a model should imply that a better outcome is achieved, as more information is available. However, this is not always the case, as some features may be redundant or irrelevant to discriminate glaucoma. These features may decrease the accuracy and increase training time. Hence, a feature selection algorithm based on univariate analysis of the features was applied. The method consisted of applying a statistical test to compare the discriminating power of each feature and hence retain the subset of the k features that presented the lowest *P* values. For the binary classification, the Mann-Whitney test's *P* value was computed between each feature and the class labels. The Mann-Whitney test was replaced by the Kruskal-Wallis test for the multiclass classification (healthy, mild, moderate, and severe). For each possible value of k, the cross-validation accuracy estimate within the training set was used as a criterion to be maximized during the hyperparameter optimization. The optimal value of k was determined on the test set by selecting the value that maximized the average accuracy over the 121 outer cross-validation folds.

#### Statistical Analysis and Feature Study

The normal distribution of the data on each studied group was assessed with the Kolmogorov-Smirnov test. The Mann-Whitney test was used to compare the statistical differences for all features between control and glaucoma and between POAG and NTG subjects. The severity levels of glaucoma were compared with the one-way analysis permutation test for all features followed by a post hoc analysis using the pairwise permutation test with false discovery rate adjustment. The AUROC, accuracy, sensitivity, and specificity of the classification between control and glaucoma subjects, as well as types of glaucoma, were computed for each model. The 95% confidence interval of each metric was obtained with stratified bootstrap resampling.^48^ The comparison between the ROC curves from the binary classification models was performed with the DeLong test,^49^^,^^50^ whereas the comparison between contingency tables for the multiclass models was performed with the McNemar-Bowker test.^51^^,^^52^ For all tests, P<0.05 was used to declare significance. The multiclass microaveraged AUROC and accuracy were used to assess the performance of the multiclass models (i.e., to classify between the four glaucoma severity levels: healthy, mild, moderate, and severe).^39^^,^^53^

## Results

The demographics and characteristics of all subjects included in the data set are listed in Table 1. No statistically significant age differences were observed between the healthy control and the glaucoma groups (P=0.1), type of glaucoma (P=0.2), or severity groups (P=0.74). A statistically significant difference was observed for the VF MD between the control and glaucoma groups (P<0.001) and between the severity groups (P<0.001) but not between the POAG and NTG groups (P=0.49). The RNFL thickness was significantly different between the control and glaucoma groups but not between POAG and NTG groups (P=0.2). A significant difference was also observed between the three severity groups (P<0.001) for the RNFL. However, the post hoc analysis revealed that no significant differences existed between the moderate and severe groups (P=0.23). The inferotemporal microvascular density of the superficial layer was not significant to differentiate between POAG and NTG (P=0.77) but presented a statistically significant difference between control and glaucoma groups (P<0.001) and among severity groups (P<0.001), including between moderate and severe glaucoma (P=0.034).

### Glaucoma Classification

**Figure 7.**
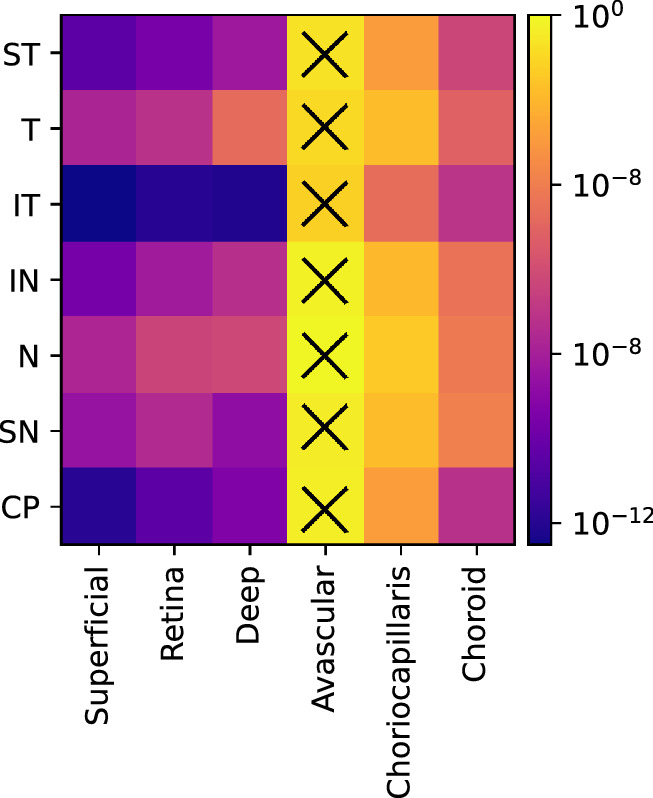
Mann-Whitney test, *p* value, for each vascular feature between control and glaucoma subjects. The X symbol denotes the groups that did not present a statistically significant difference (*P* >0.05).

**Figure 8.**
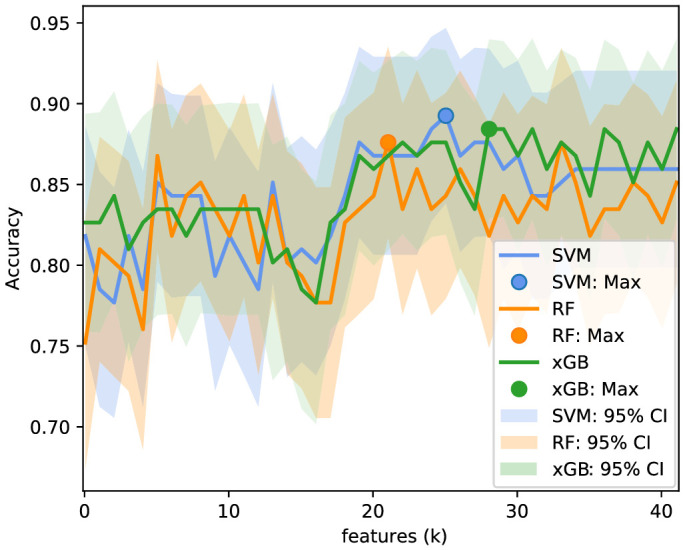
Accuracy for the selected features for each subset size k. The same procedure was applied to all models, SVM (*blue*), RF (*orange*), and xGB (*green*), for classifying between the healthy control and glaucoma groups.

The Garway-Heath sectorial analysis of the six layers of the peripapillary region resulted in 42 microvascular features. Figure 7 shows the logarithm of the Mann-Whitney test, *P* value, between control and glaucoma subjects for each feature, color-coded by significance level. All features except the avascular layer for all sectors presented a statistically significant difference (P<0.05) between glaucoma and controls. The IT sector had the lowest *P* values, in agreement with what has been reported in the literature. The results of the classification (AUROC, accuracy, sensitivity, and specificity) between glaucoma and healthy individuals before and after feature selection (according to the method described in Classification) are summarized in Table 2. For both models, a reduction of the number of features was observed (42 to 26, 22, and 29 for SVM, RF, and xGB, respectively). Figure 8 shows the accuracy as a function of the selected number of features (k). The selected features based on the highest accuracy observed in Figure 8 are listed in Table 3. Although the optimal number of features differed between models, an increase of the accuracy can be observed in the three models when increasing the number of features. Since the MLS SVM after feature selection presented the highest score in AUROC and accuracy, it was considered the best model for glaucoma detection.

**Table 2.** AUROC, Accuracy, Sensitivity, and Specificity (± Confidence Interval at 95%) for the Vascular and Structural Parameters before and after Feature Selection for the Classification between Glaucoma and Healthy Individuals

			Before Feature Selection			
Parameter	Model	No. of Features	AUROC	Accuracy	Sensitivity	Specificity
Vascular	S-IT SVM	1	0.81 ± 0.07	0.82 ± 0.07	0.83 ± 0.08	0.79 ± 0.13
	S-IT RF	1	0.76 ± 0.08	0.75 ± 0.08	0.73 ± 0.10	0.79 ± 0.13
	S-IT xGB	1	0.81 ± 0.08	0.83 ± 0.07	0.87 ± 0.07	0.74 ± 0.14
	MLS SVM	42	0.87 ± 0.06	0.86 ± 0.06	0.84 ± 0.08	0.90 ± 0.09
	MLS RF	42	0.80 ± 0.08	0.81 ± 0.07	0.83 ± 0.08	0.77 ± 0.13
	MLS xGB	42	0.84 ± 0.07	0.86 ± 0.06	0.90 ± 0.06	0.77 ± 0.13
Structural	RNFL SVM	1	0.85 ± 0.06	0.82 ± 0.07	0.76 ± 0.09	**0.95 ± 0.07**
	RNFL RF	1	0.82 ± 0.07	0.80 ± 0.07	0.77 ± 0.09	0.87 ± 0.11
	RNFL xGB	1	0.82 ± 0.07	0.82 ± 0.07	0.82 ± 0.08	0.82 ± 0.12
			After Feature Selection			
Vascular	MLS SVM	26	**0.89 ± 0.06**	**0.90 ± 0.05**	0.91 ± 0.06	0.87 ± 0.10
	MLS RF	22	0.86 ± 0.06	0.87 ± 0.06	0.88 ± 0.07	0.85 ± 0.11
	MLS xGB	29	0.85 ± 0.07	0.88 ± 0.06	**0.93 ± 0.05**	0.77 ± 0.13

The highest mean value per metric is highlighted in bold.

**Table 3.** List of Features for the SVM, RF, and xGB Models Used to Train the Final Multilayer and Multisector Models for Classifying Control and Glaucoma Subjects

**SVM**				**RF**				**xGB**			
Layer	Sector	$log_{10}\left(p\right)$	Folds	Layer	Sector	$log_{10}\left(p\right)$	Folds	Layer	Sector	$log_{10}\left(p\right)$	Folds
Superficial	IT	−12.6	121	Superficial	IT	−12.6	121	Superficial	IT	−12.6	121
Deep	IT	−12.0	121	Deep	IT	−12.0	121	Deep	IT	−12.0	121
Retina	IT	−11.9	121	Retina	IT	−11.9	121	Retina	IT	−11.9	121
Superficial	CP	−11.8	121	Superficial	CP	−11.8	121	Superficial	CP	−11.8	121
Superficial	ST	−10.3	121	Superficial	ST	−10.3	121	Superficial	ST	−10.3	121
Retina	CP	−10.3	121	Retina	CP	−10.3	121	Retina	CP	−10.3	121
Superficial	IN	−9.5	121	Superficial	IN	−9.5	121	Superficial	IN	−9.5	121
Retina	ST	−9.4	121	Retina	ST	−9.4	121	Retina	ST	−9.4	121
Deep	CP	−9.2	121	Deep	CP	−9.2	121	Deep	CP	−9.2	121
Deep	SN	−8.7	121	Deep	SN	−8.7	121	Deep	SN	−8.7	121
Superficial	SN	−8.4	121	Superficial	SN	−8.4	121	Superficial	SN	−8.4	121
Deep	ST	−8.2	121	Deep	ST	−8.2	121	Deep	ST	−8.2	121
Retina	IN	−8.1	121	Retina	IN	−8.1	121	Retina	IN	−8.1	121
Superficial	T	−7.7	121	Superficial	T	−7.7	121	Superficial	T	−7.7	121
Superficial	N	−7.6	121	Superficial	N	−7.6	121	Superficial	N	−7.6	121
Retina	SN	−7.5	121	Retina	SN	−7.5	121	Retina	SN	−7.5	121
Deep	IN	−7.2	121	Deep	IN	−7.2	121	Deep	IN	−7.2	121
Choroid	CP	−7.2	121	Choroid	CP	−7.2	121	Choroid	CP	−7.2	121
Retina	T	−7.1	121	Retina	T	−7.1	121	Retina	T	−7.1	121
Choroid	IT	−7.0	121	Choroid	IT	−7.0	121	Choroid	IT	−7.0	121
Retina	N	−6.3	121	Retina	N	−6.3	120	Retina	N	−6.3	121
Choroid	ST	−6.2	121	Choroid	ST	−6.3	93	Choroid	ST	−6.2	121
Deep	N	−6.1	121	Deep	N	−6.3	29	Deep	N	−6.1	121
Choroid	T	−5.0	121					Choroid	T	−5.0	121
Deep	T	−4.7	116					Deep	T	−4.7	121
Choriocapillaris	IT	−4.6	110					Choriocapillaris	IT	−4.6	121
Choroid	IN	−4.6	15					Choroid	IN	−4.4	121
Choroid	SN	−4.3	1					Choroid	N	−4.1	121
								Choroid	SN	−3.9	121

The selected features are ranked from the top to the bottom with the $log_{10}\left(p\right)$ as the averaged logarithm value over the folds in which the feature was selected.

Further analysis of the ROC curves showed a statistically significant difference (P=0.02) between one single feature (S-IT SVM) and the model after feature selection (MLS SVM). However, no statistically significant difference was observed (P=0.24) between the best structural model (RNFL SVM) and the best vascular model (MLS SVM).

### Type of Glaucoma

The results of the classification between the two types of glaucoma, POAG and NTG, are shown in Table 4. The accuracy was maximized at selecting 4, 6, and 38 features for the MLS SVM, MLS RF, and MLS xGB model, respectively. However, in none of the cases, neither before nor after feature selection, a high discrimination ability was observed. The structural model (RNFL RF) presented the highest sensitivity, although, as shown in Table 1, no statistically significant difference existed between both groups (POAG versus NTG, P=0.20).

**Table 4.** AUROC, Accuracy, Sensitivity, and Specificity (± Confidence Interval at 95%) for the Vascular and Structural Parameters before and after Feature Selection for Classifying POAG and NTG Patients

			Before Feature Selection			
Parameter	Model	No. of Features	AUROC	Accuracy	Sensitivity	Specificity
Vascular	S-IT SVM	1	0.11 ± 0.07	0.11 ± 0.07	0.14 ± 0.10	0.08 ± 0.09
	S-IT RF	1	0.13 ± 0.07	0.12 ± 0.07	0.00 ± 0.00	0.26 ± 0.14
	S-IT xGB	1	0.32 ± 0.08	0.34 ± 0.11	0.59 ± 0.15	0.05 ± 0.07
	MLS SVM	42	0.49 ± 0.11	0.49 ± 0.11	0.43 ± 0.14	0.55 ± 0.16
	MLS RF	42	0.45 ± 0.10	0.45 ± 0.10	0.45 ± 0.14	0.45 ± 0.16
	MLS xGB	42	0.44 ± 0.11	0.45 ± 0.11	0.55 ± 0.15	0.34 ± 0.16
Structural	RNFL SVM	1	**0.61 ± 0.11**	0.61 ± 0.11	0.59 ± 0.14	0.63 ± 0.15
	RNFL RF	1	**0.61 ± 0.10**	**0.62 ± 0.11**	**0.77 ± 0.13**	0.45 ± 0.16
	RNFL xGB	1	0.49 ± 0.11	0.49 ± 0.11	0.52 ± 0.15	0.45 ± 0.16
			After Feature Selection			
Vascular	MLS SVM	4	0.57 ± 0.10	0.56 ± 0.11	0.45 ± 0.15	**0.68 ± 0.15**
	MLS RF	6	0.51 ± 0.11	0.51 ± 0.11	0.55 ± 0.15	0.47 ± 0.16
	MLS xGB	38	0.46 ± 0.11	0.46 ± 0.11	0.50 ± 0.15	0.42 ± 0.16

The highest mean value per metric is highlighted in bold.

### Glaucoma Severity

**Figure 9.**
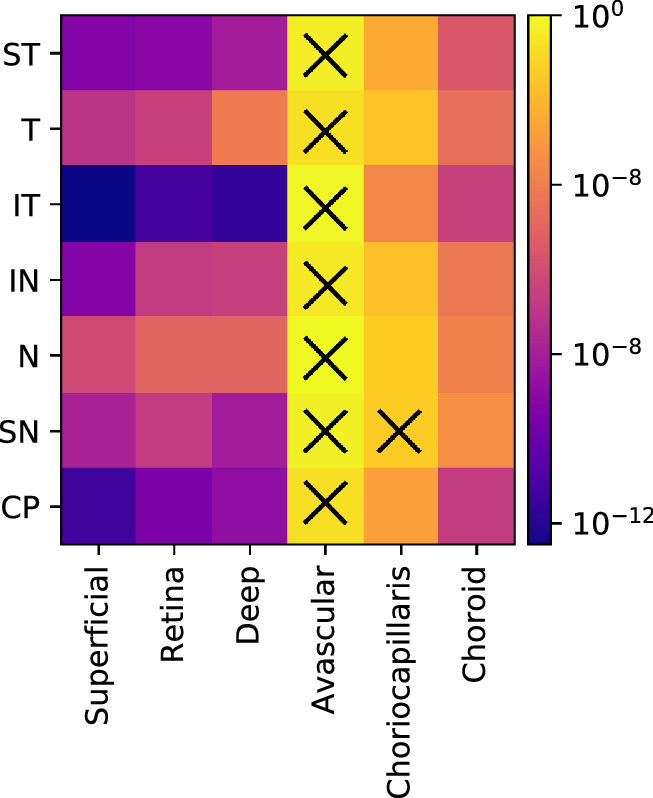
Permutation test, *p* value, for each vascular feature between severity groups (healthy controls, mild, moderate, and severe glaucoma). The X symbol denotes the groups that did not present a statistically significant difference (*P* >0.05).

Figure 9 shows the logarithm of the permutation test for one-way analysis, $log_{10}$(*P* value), between glaucoma severity groups, including the healthy controls for each feature, color-coded by significance level. All features except the avascular layer (for all sectors) and the choriocapillaris (SN sector) presented a statistically significant difference (P<0.05) between groups. The inferotemporal (at the superficial, retina, and deep layers) and circumpapillary sectors (at the superficial layer) were the most discriminant for severity classification.

**Figure 10.**
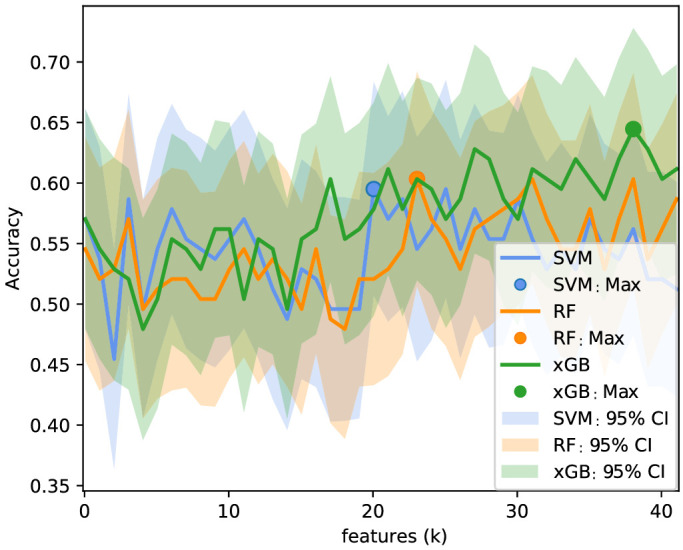
Accuracy for the selected features for each subset size *k*. The same procedure was applied to all models, SVM (*blue*), RF (*orange*), and xGB (*green*), for classifying the severity groups.

Table 5 summarizes the AUROC, accuracy, sensitivity, and specificity for all multiclass models before and after feature selection. The accuracy as a function of the number of features retrieved from the feature selection step, as well as the respective ranked features, is shown in Figure 10 and Table 6, respectively. In interpreting these results, please note that the multi-class accuracy measure for this four-class classification scenario would be 0.25 in case of complete random guessing; a value of 0.5 to 0.6 therefore indicates good classification accuracy. Among the compared models, the MLS xGB after feature selection was the most discriminant for the multiclass comparison. The accuracy slightly improved after feature selection, although no statistically significant difference was observed according to the McNemar-Bowker test (P=0.57).

**Table 5.** AUROC and Accuracy (± Confidence Interval at 95%) for the Vascular and Structural Parameters before and after Feature Selection for Classifying Healthy Controls and Different Glaucoma Severity Levels (Mild, Moderate, and Severe)

			Before Feature Selection			
Parameter	Model	No. of Features	AUROC	Accuracy	Sensitivity	Specificity
Vascular	S-IT SVM	1	0.72 ± 0.06	0.58 ± 0.09	0.58 ± 0.09	0.86 ± 0.04
	S-IT RF	1	0.70 ± 0.06	0.55 ± 0.09	0.55 ± 0.09	0.84 ± 0.04
	S-IT xGB	1	0.72 ± 0.06	0.58 ± 0.09	0.58 ± 0.09	0.85 ± 0.04
	MLS SVM	42	0.67 ± 0.06	0.51 ± 0.09	0.51 ± 0.09	0.83 ± 0.04
	MLS RF	42	0.72 ± 0.06	0.60 ± 0.09	0.60 ± 0.09	0.85 ± 0.04
	MLS xGB	42	0.72 ± 0.06	0.59 ± 0.09	0.59 ± 0.09	0.85 ± 0.04
Structural	RNFL SVM	1	0.60 ± 0.06	0.40 ± 0.09	0.40 ± 0.09	0.79 ± 0.04
	RNFL RF	1	0.59 ± 0.06	0.39 ± 0.09	0.39 ± 0.09	0.80 ± 0.03
	RNFL xGB	1	0.67 ± 0.06	0.53 ± 0.09	0.53 ± 0.09	0.82 ± 0.04
			After Feature Selection			
Vascular	MLS SVM	21	0.74 ± 0.06	0.60 ± 0.09	0.60 ± 0.09	**0.87 ± 0.04**
	MLS RF	24	0.69 ± 0.06	0.55 ± 0.09	0.55 ± 0.09	0.84 ± 0.04
	MLS xGB	39	**0.76± 0.06**	**0.64 ± 0.08**	**0.64 ± 0.08**	**0.87 ± 0.04**

The highest mean value per metric is highlighted in bold.

**Table 6.** List of Features for the SVM and RF Models Used to Train the Final Multilayer and Multisector Models for Classifying the Severity Groups Including the Healthy Controls

**SVM**				**RF**				**xGB**			
Layer	Sector	$log_{10}\left(p\right)$	Folds	Layer	Sector	$log_{10}\left(p\right)$	Folds	Layer	Sector	$log_{10}\left(p\right)$	Folds
Superficial	IT	−14.9	121	Superficial	IT	−14.9	121	Superficial	IT	−14.9	121
Retina	IT	−14.0	121	Retina	IT	−14.0	121	Retina	IT	−14.0	121
Deep	IT	−13.9	121	Deep	IT	−13.9	121	Deep	IT	−13.9	121
Superficial	CP	−13.7	121	Superficial	CP	−13.7	121	Superficial	CP	−13.7	121
Superficial	IN	−12.5	121	Superficial	IN	−12.5	121	Superficial	IN	−12.5	121
Superficial	ST	−11.7	121	Superficial	ST	−11.7	121	Superficial	ST	−11.7	121
Retina	CP	−11.6	121	Retina	CP	−11.6	121	Retina	CP	−11.6	121
Retina	IN	−11.0	121	Retina	IN	−11.0	121	Retina	IN	−11.0	121
Deep	CP	−10.9	121	Deep	CP	−10.9	121	Deep	CP	−10.9	121
Deep	IN	−10.7	121	Deep	IN	−10.7	121	Deep	IN	−10.7	121
Retina	ST	−10.1	121	Retina	ST	−10.1	121	Retina	ST	−10.1	121
Deep	ST	−10.1	121	Deep	ST	−10.1	121	Deep	ST	−10.1	121
Deep	SN	−9.7	121	Deep	SN	−9.7	121	Deep	SN	−9.7	121
Superficial	SN	−9.6	121	Superficial	SN	−9.6	121	Superficial	SN	−9.6	121
Superficial	N	−8.8	121	Superficial	N	−8.8	121	Superficial	N	−8.8	121
Superficial	T	−8.5	121	Superficial	T	−8.5	121	Superficial	T	−8.5	121
Retina	SN	−8.3	121	Retina	SN	−8.3	121	Retina	SN	−8.3	121
Deep	N	−7.1	121	Deep	N	−7.1	121	Deep	N	−7.1	121
Retina	N	−7.1	121	Retina	N	−7.1	121	Retina	N	−7.1	121
Retina	T	−7.1	121	Retina	T	−7.1	121	Retina	T	−7.1	121
Choroid	IT	−6.3	120	Choroid	IT	−6.3	121	Choroid	IT	−6.3	121
Choroid	CP	−6.3	1	Choroid	CP	−5.9	121	Choroid	CP	−5.9	121
				Choriocapillaris	IT	−5.0	121	Choriocapillaris	IT	−5.0	121
				Choroid	ST	−4.9	121	Choroid	ST	−4.9	121
								Deep	T	−4.3	121
								Choroid	T	−4.2	121
								Choroid	IN	−3.1	121
								Choroid	N	−3.0	121
								Choroid	SN	−3.0	121
								Choriocapillaris	CP	−2.1	121
								Choriocapillaris	ST	−1.9	121
								Choriocapillaris	T	−1.7	121
								Choriocapillaris	IN	−1.2	121
								Choriocapillaris	SN	−1.2	121
								Choriocapillaris	N	−0.9	121
								Avascular	IT	−0.9	121
								Avascular	CP	−0.5	120
								Avascular	IN	−0.4	117
								Avascular	T	−0.3	115
								Avascular	ST	−0.3	10
								Avascular	SN	−0.3	1

The selected features are ranked from the top to the bottom with the $log_{10}\left(p\right)$ as the averaged logarithm value over the folds in which the feature was selected.

**Figure 11.**
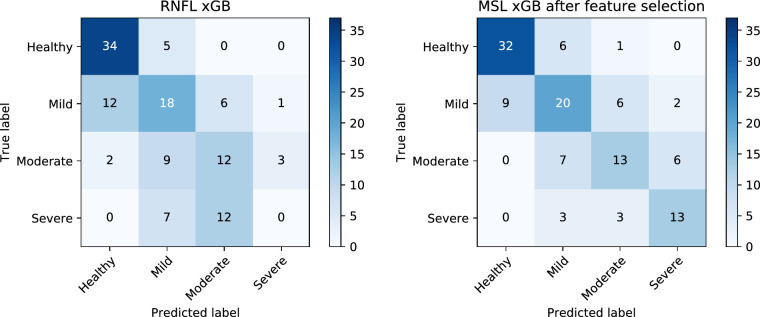
Confusion matrices for each severity group according to the best structural (RNFL xGB model, *left*) and the best vascular model (MSL xGB model after feature selection, *right*).

Figure 11 shows the confusion matrices for the best structural (RNFL xGB) and vascular (MSL xGB after feature selection) models. In both cases, the advanced stages of glaucoma were challenging to classify, with the RNFL xGB misclassifying all the severe cases. It can be observed from the confusion matrices that the RNFL xGB performed better for the healthy controls. However, the results of Glaucoma Classification showed that there was no statistical difference between the RNFL and the best vascular density model on classifying healthy and glaucoma subjects. The vascular model had a better performance discriminating between different glaucoma severity levels: the confusion matrices show that it was more likely to classify the right class but also that mistakes were more likely to happen in consecutive classes (i.e., mistake severe for moderate rather than mistake severe for mild). Moreover, a statistically significant difference was observed between models based on the McNemar-Bowker test (*P* = 0.01), confirming that, overall, the vascular information outperformed the structural information at discriminating different glaucoma severity groups.

## Discussion

A number of approaches and techniques have been published over the past two decades to infer and study vascular glaucomatous damage.^26^ OCTA is a new submodality that has emerged from OCT, which measures changes in backscattered signal intensity or amplitude in order to differentiate areas of blood flow from areas of static tissue. OCTA requires a very high sampling density in order to achieve the resolution needed to detect the tiny capillaries found in the retina. Its current limitations include inability to show leakage, proclivity for image artifacts due to patient movement/blinking,^54^ and a relatively small field of view (e.g., 3 x 3 mm, 4.5 x 4.5 mm, and 6 x 6 mm). Hence, studies incident on glaucoma diagnosis and progression have been focused on specific regions of interest, namely the macula and the optic disc, including the peripapillary region. Richter et al.,^55^ reported that, although the superficial vascular plexus microcirculation in both macular and peripapillary regions is significantly reduced in glaucoma patients, global peripapillary perfusion parameters outperform global macular perfusion parameters. The same observations were reported by Triolo et al.,^56^ and Rao et al.,^23^ which led to the conclusion that the peripapillary region should provide the highest discriminant power to classify glaucoma. Although the OCTA data, in their raw form, are provided in three dimensions, the two-dimensional data created from projections between specific depths are often used. Coronal projections of different layers (superficial vascular plexus,^31^^,^^57^^,^^58^ deep vascular plexus,^31^^,^^59^^,^^60^ whole retina,^21^^,^^61^ choriocapillaris,^61^ and choroid^61^^,^^62^) have been studied individually. However, to the best of our knowledge, no one has reported whether including information from multiple layers at the peripapillary region has an added value for the glaucoma classification.

Three classification models (SVM, RF, and xGB) were used in this work. All models are relatively fast and appropriate for small data sets and high-dimensional feature spaces. However, there are a number of other suitable options that could have been used in this work, such as KNN, or decision trees.^63^ Besides conventional machine learning models, deep learning approaches can be used in classification tasks and have become the state of the art in several medical imaging applications. The main drawback when training a deep learning model is that it requires large amounts of data, which were not available in this study. Nevertheless, a convolutional neural network (CNN) model (VGG16,^64^) was explored to compare with the proposed methods in all three classification tasks. Pretrained weights and data augmentation were used to minimize the issues derived from the small data set size. Unfortunately, the CNN was not able to outperform the traditional classifiers in any of the cases. The implementation details and results of this approach are detailed in the Appendix.

In Glaucoma Classification, we show that combining the information from multiple layers and multiple sectors at the peripapillary region has an added value for glaucoma classification, in comparison to using only the most discriminant region (superficial layer, inferotemporal sector). The comparison of healthy individuals with glaucoma patients showed that it is possible to achieve a classification accuracy using vascular information similar to that obtained with structural information (statistically nonsignificant differences). The MLS SVM obtained the best results among the compared models to differentiate healthy from glaucoma individuals, although the differences with the RNFL were not significant. The differences between using the most discriminant feature for microvascular analysis, the inferotemporal sector in the superficial layer, and using the feature selection subset were significant, showing that there is an added value of using different layers and sectors for glaucoma classification. However, it is difficult to conclude whether these differences are from the pathology itself or a consequence of the imaging artifacts. In general, deeper layers have not been considered in glaucoma analysis due to the difficulty in explaining the physical meaning of the imaged content. Deeper layers are influenced by light propagation through the overlying vessels, which cause the projection of intensity and phase fluctuations into deeper layers. These are known as shadowing artifacts. Although latest algorithms provided by the manufacture tend to minimize these influences, residual artifacts remain. From the univariate analysis, it is also possible to observe that the superficial is the most informative layer for discriminating glaucoma, in agreement with what has been reported in the literature. Moreover, looking at the sectors, it can be confirmed that the inferotemporal sector is the most affected by the disease, following also what has already been published about typical locations for optic nerve damage.^65^ Overall, the obtained results regarding the most informative feature and accuracy of the models are in agreement with the latest analysis of the peripapillary region.^23^^,^^31^^,^^66^

Only a few studies have analyzed the differences between POAG and NTG. Bojikian et al.,^67^ observed that the perfusion detected on the optic disc was significantly reduced in POAG and NTG groups compared to normal controls, but no difference was seen between POAG and NTG groups with similar levels of VF MD damage. In this study, an attempt to differentiate both groups was also made. However, the results show that, regardless of the type of parameters used (structural or vascular), the model (SVM, RF, or xGB), and the application or not of feature selection, no differentiation could be made in this data set. This can be due to the data set not being sufficiently large or representative to obtain conclusions, but it can also point to the impossibility of making a distinction between POAG and NTG based on a single, static OCTA analysis. Since many studies have shown that NTG patients are more prone to vascular dysregulation, it is possible that these differences are clearer in a dynamic (rather than static) examination of ONH-OH/retinal vasculature, comparing OCTA data obtained through several acquisitions in the same patient.

The glaucoma group was subdivided into three groups according to the severity (mild, moderate, and severe). The MLS xGB model achieved the best outcomes for accuracy in the multiclass analysis. From the multiclass confusion matrices in Figure 11, it was possible to observe that the best vascular model classifies the severity level correctly more often than the structural model, especially in the intermediate levels. The poorer performance of the structural parameters compared to the vascular analysis may be explained by the floor effect in the RNFL as the disease progresses. Structural parameters measured by OCT reach a base level beyond which little change was seen with increasing severity of glaucoma.^68^ The average value generally lies between 50% and 70% of the RNFL in normal eyes.^69^^-^^71^

Despite the results observed in this study, further research needs to be done in order to reduce the image variability due to the image acquisition and post-processing. Since there is not a clear boundary that can be applied to differentiate micro- from macrovasculature in OCTA imaging, different image- processing approaches may lead to different microvascular interpretations within the same data. It is a subjective definition that may be interpreted differently depending on the image operator. A popular method used to extract the microvascular density is to generate a binarization from the OCTA image, based on thresholding techniques.^72^ The ratio of white or black pixels over a specific area is used to estimate the microvascular dropout. In general, the threshold is chosen based on an empirical analysis using general-purpose image-processing programs such as ImageJ.^73^ These binarization approaches lead to results that are valid for a specific method, which ultimately hinder comparisons between different studies, and preclude the development of a robust classification model. In this study, it is assumed that the OCTA images exported from a single device are comparable between subjects and, hence, the median serves as a robust measure to estimate the microvascular density within a ROI, provided that the microvasculature is properly segmented. The separation of micro- from macrovasculature is another source of variability between studies. In some studies, the macrovasculature is segmented and extracted from the region of interest. Other authors have opted for estimating the vascular density based on all the information presented on the OCTA image. Since the macrovasculature is not expected to be affected by glaucoma and is a subject-dependent anatomic feature, an analysis including the macrovasculature based on image pixel intensity is not desirable, as it may bias the results.^74^ Similarly, the size of the ONH-OH is subject dependent and does not provide relevant information. Therefore, it is desirable to segment and exclude these areas from the ROI before the microvascular density estimation is done. Deep learning segmentation approaches could eventually reduce the current variability given their proven efficiency in vascular segmentation in fundus photography and scanning laser ophthalmoscopy imaging.^75^ All of these adjustments should be routinely done to guarantee more accurate (and comparable) results when analyzing the role of vascular parameters in glaucoma with OCTA.

Although this study hints that microvascular density outperforms the RNFL thickness in multiclass classification of glaucoma severity, further work on larger cohorts is needed in order to validate this hypothesis. As seen in Figures 8 and 10, the accuracy as a function of the number of features (based on the entire data set) was fluctuating substantially, and there was no clear and robust maximum in the graph. Thus, the accuracies reported after feature selection might be a bit optimistic. Future studies should also evaluate the predictive value of RNFL when using the same Garway-Heath sectors corrected for the fovea-disc axis. In addition, the prediction ability of vascular and structural information using longitudinal data should be analyzed, as well as the design of new models combining multisectorial data from both types of parameters.

## Conclusion

In order to ensure individualized health care for glaucoma patients, taking into account the disease and severity, it is necessary to develop models and software that are able to integrate the increasingly complex web of risk factors and parameters associated with the disease. Hence, studies in glaucoma research must expand beyond the established risk factors and explore the contribution of novel technologies. In this sense, the inclusion of image-based parameters, such as measurements derived from OCT and OCTA, is particularly relevant, since these have high reproducibility and are largely operator independent. In line with this research goal, this study assesses the effect of combining vascular information of different retinal and choroidal layers to improve the prediction and staging of glaucoma disease. The results show that, although the OCTA superficial vascular plexus is the most informative at discriminating healthy subjects from glaucoma patients, and also between glaucoma severity levels, there is an added value of including multilayer and multisector information in a classification model, instead of restricting the information to the most discriminative region. Moreover, a combination of multilayer and multisector microvascular information seems to yield a higher discriminative power than the circumpapillary RNFL thickness to discriminate between severity levels of glaucoma. Nevertheless, further studies must be done to better understand and validate the role of deeper layers such as choriocapillaris and choroid in glaucomatous vascular damage, as well as to reduce the bias due to image acquisition and postprocessing. In addition, multisectorial RNFL data and larger cohorts should also be considered in future research.

## Figures and Tables

**Figure A1.**
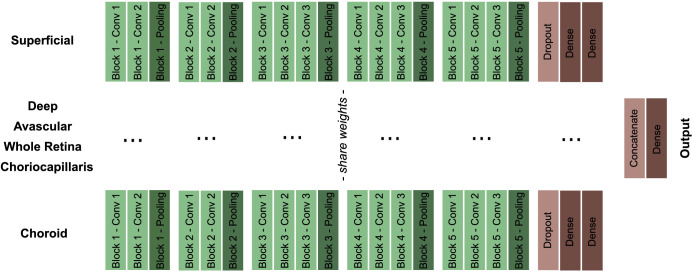
VGG16 architecture modified for each of the six OCTA images. The weights of the convolutional blocks (in *green*) were freezed, while the dense layers were trained.

**Figure A2.**
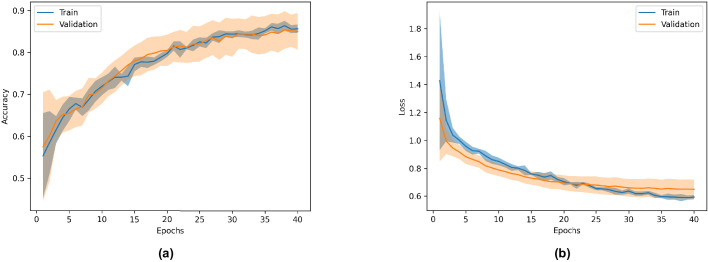
Mean and confidence interval at 95% for the accuracy (a) and loss (b) of the model trained to classify healthy controls versus glaucoma subjects.

## Appendix

In this section, we summarize the obtained results for the classification of glaucoma disease (healthy, glaucoma), type of glaucoma (POAG, NTG), and glaucoma severity (healthy, mild, moderate, and severe) using deep learning (DL).^76^ Among the different possibilities, a transfer-learning approach based on a CNN classification model was applied. In transfer learning, a new task is learned through the transfer of knowledge from a task that has already been learned.^77^ The task used to train the model does not need to be related to the objective task. Such a technique reduces the amount of data required and, in many cases, increases the accuracy as compared to models built from scratch.

### Methods

A state-of-the-art architecture for classification problems, VGG16,^64^ was used as basis for the model. All OCTA images were resized to match the VGG16's expected input (224 × 224 × 3). Since the current data set consists of six vascular layers per subject (see Fig. 2), a VGG16 model was implemented per layer. The weights of the six models were shared, as otherwise the number of trainable parameters would be too high, potentially leading to overfitting. Regarding each individual VGG16, the convolutional blocks were kept as in the original architecture, but the last block, which consists of three fully connected layers, was altered to reduce the model complexity and to prevent overfitting (see Fig. A1). A spatial dropout^78^ layer of 0.25 was added to the block, and the number of neurons of the two fully connected layers was reduced from 4096 to 2048. A concatenation layer was used to merge the output of all six models, and the number of neurons of the output layer was changed from 1000 to 2 and 4 for the binary and multiclass classification, respectively. The network was initialized with the ImageNet^79^ weights, and only the fully connected layers were retrained. Tensorflow^80^ and Keras^81^ libraries were used for the implementation of the models.

To prevent the model from overfitting the training set and to improve its generalization capabilities, data augmentation was performed, increasing the number of samples in the data set by a factor of 5. The following ranges of transformation and normalization operations were applied to the data: rotation (up to 10 degrees), height and width shift (ranging up to 10%), horizontal flip, zoom, and brightness (ranging from 80% to 120%).

Experiments with various hyperparameter values were conducted to maximize the performance of the CNN model for classifying healthy controls against glaucoma subjects. The optimizer was the stochastic gradient descent that adapts learning rates based on a moving window of gradient updates (Adadelta).^82^ The binary cross-entropy was chosen as loss function, and the training ran for 40 epochs with a batch size of 16. The hyperparameters chosen for this first classification task were also used for training the models to classify POAG versus NTG and glaucoma severity levels. For the latter, the categorical cross-entropy loss function was used. Given the constraints in computing power, stratified cross-validation was used to split the data in five folds, instead of the leave-one-out pipeline used in the classical methods. The number of images for each class was the same for each fold.

### Results and Discussion

Figure A2 shows the mean and confidence interval at 95% for the accuracy (a) and loss (b) of the model trained to classify healthy controls versus glaucoma subjects. The AUROC, accuracy, sensitivity, and specificity for the three models are summarized in Table A1.

**Table A1.** AUROC, Accuracy, Sensitivity, and Specificity (± Confidence Interval at 95%) for the Glaucoma Classification, Type of Glaucoma, and Glaucoma Severity Analysis Based on the Transfer Learning Approach

**Analysis**	**AUROC**	**Accuracy**	**Sensitivity**	**Specificity**
Glaucoma classification *(healthy, glaucoma)*	0.83±0.05	0.87±0.03	0.73±0.10	0.94±0.03
Type of glaucoma *(POAG, NTG)*	0.65±0.09	0.65±0.09	0.65±0.19	0.66±0.13
Glaucoma severity *(healthy, mild, moderate, severe)*	0.63±0.03	0.47±0.05	0.47±0.05	0.82±0.02

The results show that the presented transfer learning approach does not outperform the best conventional models presented earlier in this work. For the healthy control versus glaucoma subjects, the specificity was better than the best model presented in Table 2. However, the AUROC, the accuracy, and the sensitivity presented lower values. The model trained to classify the type of glaucoma did present slightly better results for the AUROC, accuracy, and specificity than the best model in Table 4. However, no significant statistical difference was observed between the ROC curves (P=0.46). Moreover, the results are still underwhelming, and it is not possible to claim that OCTA data can be used to differentiate between POAG and NTG subjects. Lastly, the model trained to differentiate between glaucoma severity levels presented lower values for all metrics when compared to the best model in Table 5. This may be explained by the low number of images of the highest severity level, as depicted in Table 1.

### Conclusion

The application of the CNN model presented in this Appendix did not show added benefits when compared to the best models presented in Tables 2, 4, and 5. Although we can not claim that other deep learning approaches could not give better results, it is certain that in-depth research would be needed to significantly improve the performance of the current classification given the size of the OCTA data set. Nevertheless, larger data sets would likely benefir from the performance of deep learning-based models.
